# Multi-datasets for different keyboard key sound recognition

**DOI:** 10.1016/j.dib.2024.110949

**Published:** 2024-09-19

**Authors:** Karwan M. Hama Rawf, Ayub O. Abdulrahman, Hana O. Kamel, Lawen M. Hassan, Ahmad O. Ali

**Affiliations:** Department of Computer Science, College of Science, University of Halabja, Halabja, Kurdistan Region, F.R. Iraq

**Keywords:** Keyboard sound classification, Acoustic side-channel attacks, Multi-datasets, Sound Recognition, Signal Processing, Social media platforms, Keyboard security

## Abstract

Keyboard acoustic recognition is a pivotal area within cybersecurity and human-computer interaction, where the identification and analysis of keyboard sounds are used to enhance security measures. The performance of acoustic-based security systems can be influenced by factors such as the platform used, typing style, and environmental noise. To address these variations and provide a comprehensive resource, we present the Multi-Keyboard Acoustic (MKA) Datasets. These extensive datasets, meticulously gathered by a team in the Computer Science Department at the University of Halabja, include recordings from six widely-used platforms: HP, Lenovo, MSI, Mac, Messenger, and Zoom. The MKA datasets have structured data for each platform, including raw recordings, segmented sound files, and matrices derived from these sounds. They can be used by researchers in keylogging detection, cybersecurity, and other fields related to acoustic emanation attacks on keyboards. Moreover, the datasets capture the intricacies of typing behaviour with both hands and all ten fingers by carefully segmenting and pre-processing the data using the Praat tool, thus ensuring high-quality and dependable data. This comprehensive approach allows researchers to explore various aspects of keyboard sound recognition, contributing to the development of robust recognition algorithms and enhanced security measures. The MKA Datasets stand as one of the largest and most detailed datasets in this domain, offering significant potential for advancing research and improving defences against acoustic-based threats.

Specifications table

**Table:** 

Subject	Sound Keyboard Acoustic Recognition, Artificial Intelligent, Signal Processing, Multimedia, Security.
Specific subject area	This combination of datasets was specifically designed for the identification and examination of multi-data related to the recognition of various keyboard key sounds. They serve as a great resource for researchers, allowing them to investigate security variation strategies across different platforms.
Type of data	Raw Data, Segmented Data by Audacity and Praat, Annotation, Matrix, Sound (wave), Matrix (MFCC)
Data collection	The Multi-Keyboard Acoustic (MKA) Datasets consist of six commonly used platforms: HP, Lenovo, MSI, Mac, Messenger, and Zoom. We obtained recordings of a user typing English text using a highly sensitive microphone. The sounds have been converted into the wave format, with each sample having a duration of exactly one second. Furthermore, every sample has been transformed into a matrix and meta-data.
Data source location	University of Halabja, IKR/IRAQ
Data accessibility	Repository name: Data Mendeley (Multi-Keyboard Acoustic (MKA) Datasets) Data identification number: (doi: 10.17632/bpt2hvf8n3.3) Direct URL to data: https://data.mendeley.com/datasets/bpt2hvf8n3/3 Instructions for accessing these data: Rawf, Karwan Mahdi; Abdulrahman, Ayub (2024), “Multi-Keyboard Acoustic (MKA) Datasets”, Mendeley Data, V3, doi: 10.17632/bpt2hvf8n3.3 [1]
Related research article	

## 1 Value of the Data

The significance of this data is in its capacity to enhance research and development endeavours in various ways:

• Research Advancement: The data serves as a great resource for investigating keyboard acoustics, sound identification, and the potential security consequences. The dataset can be utilised by researchers to investigate innovative methods for analysing keyboard sounds, enhance the precision of recognition algorithms, and examine security weaknesses linked to keyboard acoustic emanations threats.
• Comparison and Benchmarking: The dataset can be utilised by other researchers to evaluate and compare the performance of various algorithms, models, or methodologies, hence validating their usefulness.
  Cross-Domain Application Exploration: The dataset holds potential value for researchers engaged in related domains like speech recognition, natural language processing, and machine learning. They can use the data to investigate cross-domain applications, employ transfer learning methods, or create inventive solutions that connect audio-based keyboard recognition with other domains.
• Educational Applications: The dataset is suitable for educational purposes, including instructing students on signal processing, machine learning, cybersecurity, and human-computer interaction.

## 2 Background

Recent advances in deep learning, coupled with the prevalence of microphones in personal devices and the increasing use of online services through them, have significantly amplified the threat of acoustic side-channel attacks on keyboards [2]. This rising security issue involves exploiting the physical implementation of cryptosystems rather than relying on theoretical weaknesses in their algorithms. Such attacks can extract sensitive information by analyzing sound patterns produced during typing, highlighting the need for robust defense mechanisms in the digital age [3]. Side-channel attacks (SCAs) encompass a variety of methods that involve collecting and interpreting signals emitted by a device. These attacks have been successfully executed using different types of emanations, including electromagnetic (EM) waves, power consumption, mobile sensors, and sound [4].

The concept behind creating these datasets was the growing significance of keyboard sound recognition in the fields of security and user interface. The goal of this work was to provide researchers with a comprehensive resource for studying and developing solutions for keyboard acoustic emanation threats and their associated applications. By integrating theoretical knowledge from signal processing, machine learning, and cybersecurity, the plan was to construct a dataset that includes the varied acoustic features of keyboards on multiple platforms. The purpose of publishing these datasets is to expedite progress in the field, promote cooperation among academics, and support the creation of reliable recognition systems.

## 3 Data Description

Keyboard acoustic refers to the sound produced by pressing keys on a keyboard, which can vary based on factors such as key type, keyboard design, and typing technique. Applications such as keylogging detection, user authentication, and user behaviour analysis can leverage the valuable information this acoustic output carries. By analysing keyboard acoustic signals [5], Researchers aim to develop techniques for recognizing keystrokes, identifying typing patterns, and detecting anomalous behaviour. This field encompasses various methodologies, including signal processing, machine learning, and acoustic analysis, with the ultimate goal of enhancing cybersecurity measures and improving user interaction with computing devices. The Multi-Keyboard Acoustic (MKA) Datasets are organized within a main folder named “MKA Datasets”. This section provides an overview of the structure and contents of the datasets, detailing the organization of subfolders and files.

*MKA Datasets Structure:*

• Subfolders: HP, Lenovo, MSI, Mac, Messenger, Zoom, and All Platforms
• Each subfolder corresponds to a specific platform and contains data recordings for that platform.
• Additionally, there is a folder named “All Platforms” containing aggregated data from all platforms.

*Content Overview:*

1. *Subfolder Raw Data*
  • Contains original recordings of keyboard button presses under various conditions.
  • Each class has recordings lasting >15 s, totalling 73 classes.
  • Each class contains one file representing different situations.
2. *Subfolder Sound Segment (wav):*
  • Contains segmented sound files derived from the raw data.
  • Each class consists of six one-second audio files, resulting in 438 files across all classes.
  • *File format: WAV*
3. *Subfolder Sound Segment (.matrix)*
  • Contains matrices derived from the segmented sound files.
  • Each matrix corresponds to a one-second audio segment and is associated with a specific class.
  • File format: Matrix data
4. *Subfolder Sound Segment Metadata (.txt)*
  • Contains metadata files derived from the segmented sound files.
  • Each .txt file corresponds to a one-second audio segment and provides detailed information about the segment.
  • File format: Metadata text

The details of each class within each dataset are presented in Table 1, which comprises six datasets. Each dataset contains a total of around 70 classes.

**Table 1:** Summary of contents in each folder and subfolder of the MKA Datasets.

No.	Classes	HP	Lenovo	MSI	MAC	Messenger	Zoom
1	0	✓	✓	✓	✓	✓	✓
2	1	✓	✓	✓	✓	✓	✓
3	2	✓	✓	✓	✓	✓	✓
4	3	✓	✓	✓	✓	✓	✓
5	4	✓	✓	✓	✓	✓	✓
6	5	✓	✓	✓	✓	✓	✓
7	6	✓	✓	✓	✓	✓	✓
8	7	✓	✓	✓	✓	✓	✓
9	8	✓	✓	✓	✓	✓	✓
10	9	✓	✓	✓	✓	✓	✓
11	A	✓	✓	✓	✓	✓	✓
12	B	✓	✓	✓	✓	✓	✓
13	C	✓	✓	✓	✓	✓	✓
14	D	✓	✓	✓	✓	✓	✓
15	E	✓	✓	✓	✓	✓	✓
16	F	✓	✓	✓	✓	✓	✓
17	G	✓	✓	✓	✓	✓	✓
18	H	✓	✓	✓	✓	✓	✓
19	I	✓	✓	✓	✓	✓	✓
20	J	✓	✓	✓	✓	✓	✓
21	K	✓	✓	✓	✓	✓	✓
22	L	✓	✓	✓	✓	✓	✓
23	M	✓	✓	✓	✓	✓	✓
24	N	✓	✓	✓	✓	✓	✓
25	O	✓	✓	✓	✓	✓	✓
26	P	✓	✓	✓	✓	✓	✓
27	Q	✓	✓	✓	✓	✓	✓
28	R	✓	✓	✓	✓	✓	✓
29	S	✓	✓	✓	✓	✓	✓
30	T	✓	✓	✓	✓	✓	✓
31	U	✓	✓	✓	✓	✓	✓
32	V	✓	✓	✓	✓	✓	✓
33	W	✓	✓	✓	✓	✓	✓
34	X	✓	✓	✓	✓	✓	✓
35	Y	✓	✓	✓	✓	✓	✓
36	Z	✓	✓	✓	✓	✓	✓
37	apostrophe(')	✓	✓	✓	✓	✓	✓
38	dash(-)	✓	✓	✓	☒	✓	✓
39	comma(,)	✓	✓	✓	☒	✓	✓
40	Semicolon (;)	✓	✓	✓	☒	✓	✓
41	bracketopen([)	✓	✓	✓	✓	✓	✓
42	bracketclose(])	✓	✓	✓	✓	✓	✓
43	backtick(`)	✓	✓	✓	☒	✓	✓
44	equal(=)	✓	✓	✓	✓	✓	✓
45	altL	✓	✓	✓	✓	✓	✓
46	altR	✓	✓	✓	✓	✓	✓
47	asterisk	✓	✓	✓	☒	✓	✓
48	Backslash	✓	✓	✓	✓	✓	✓
49	backspace	✓	✓	✓	☒	✓	✓
50	caps	✓	✓	✓	✓	✓	✓
51	cmdL	✓	✓	✓	✓	✓	✓
52	start	☒	☒	☒	✓	☒	☒
53	down	✓	✓	✓	✓	✓	✓
54	end	✓	✓	✓	☒	✓	✓
55	enter	✓	✓	✓	✓	✓	✓
56	esc	✓	✓	✓	✓	✓	✓
57	fn	✓	✓	✓	✓	✓	✓
58	home	✓	✓	✓	☒	✓	✓
59	Lctrl	✓	✓	✓	✓	✓	✓
60	Left	✓	✓	✓	✓	✓	✓
61	Lshift	✓	✓	✓	✓	✓	✓
62	menu	✓	☒	☒	☒	☒	☒
63	fullstop	✓	✓	✓	✓	✓	✓
64	pgdn	✓	✓	✓	☒	✓	✓
65	pgup	✓	✓	✓	☒	✓	✓
66	Rctrl	✓	✓	✓	☒	✓	✓
67	Right	✓	✓	✓	✓	✓	✓
68	Rshift	✓	✓	✓	✓	✓	✓
69	Slash	✓	✓	✓	✓	✓	✓
70	Space	✓	✓	✓	✓	✓	✓
71	Tab	✓	✓	✓	✓	✓	✓
72	up	✓	✓	✓	✓	✓	✓
73	delete	☒	☒	☒	✓	☒	☒

Based on the hypothesis that subtle differences in key clicks can be exploited, a neural network is employed to classify them, leveraging its success in similar tasks like speaker identification. Despite the fact that different keys sound similar to the human ear, our investigation reveals vulnerabilities in PC keyboards to attacks based on key sound. The Multi-Keyboard Acoustic (MKA) Datasets plays a vital role in advancing researches, offering a diverse range of keyboard sounds for comprehensive model training and evaluation, and leading to the development of robust security measures against potential acoustic-based attacks [6].

**Fig. 1:**
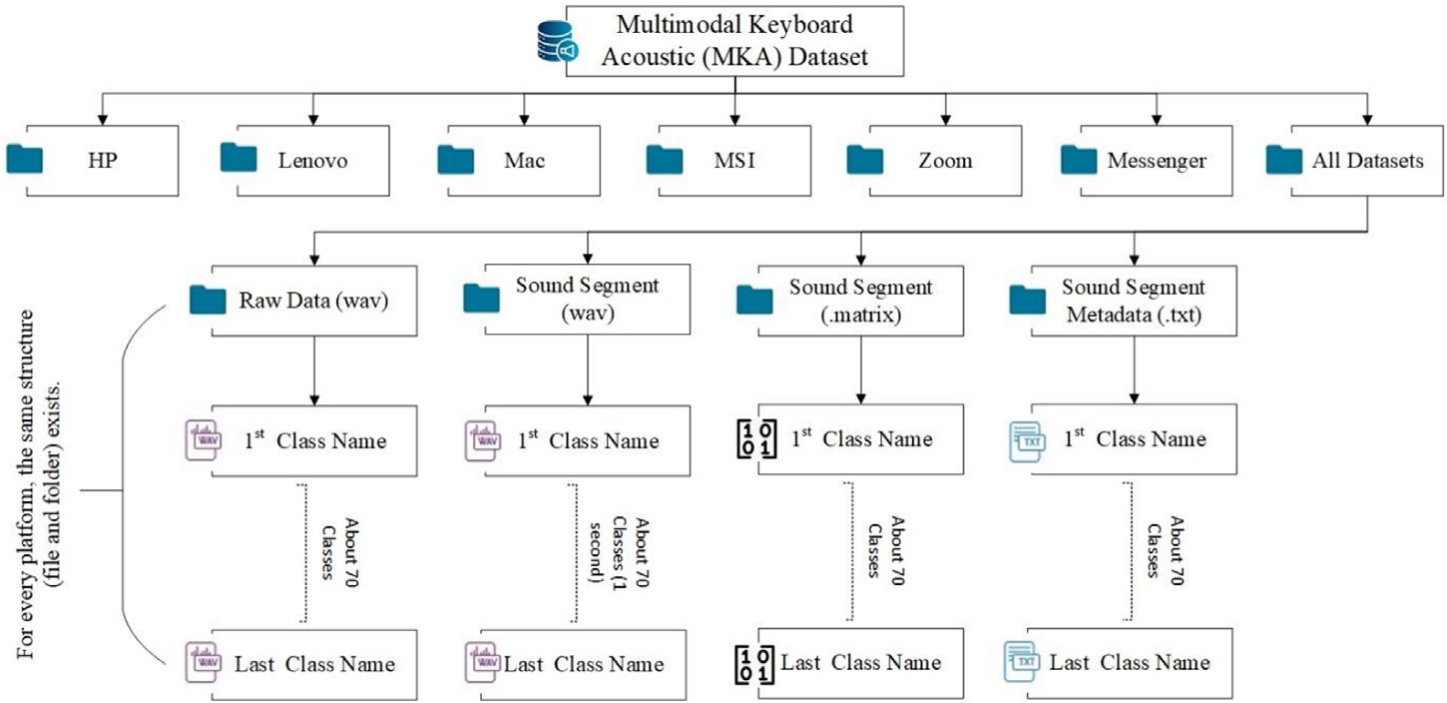
The proposed (MKA) datasets structure.

Fig. 1 illustrates the hierarchical structure of the proposed keyboard sound classification datasets. The root directory, “MKA Datasets,” encompasses seven subdirectories. Six subdirectories, named after specific platforms (hp, Lenovo, MAC, MSI, zoom, messenger), store recordings from those platforms. The seventh subdirectory, “all datasets,” combines recordings from all platforms for a comprehensive collection. Each platform subdirectory follows a consistent organization. It contains four subfolders: Raw data: This folder houses the original, unprocessed recordings for each keystroke class (detailed in Table 1). The number of files varies by platform, with around 70 files for hp, Lenovo, MSI, zoom, and messenger, and 61 files for Mac. Sound segment: This folder stores six, one-second WAV audio excerpts derived from the corresponding raw data files for each class. These files are renamed using a convention of “class_name+1” to “class_name+6” for each platform individually and “class_name+platform_name1” to “class_name+platform_name6” for all datasets. Sound segment (.matrix): This folder contains feature representations (like MFCCs) extracted from each sound segment in the corresponding “Sound segment” folder. Sound segment metadata (.txt): This folder holds detailed information associated with each sound segment, potentially including recording conditions, platform information, and keystroke class labels. The “all datasets” subdirectory mirrors this structure but contains a larger number of files per subfolder (36 files for each class in Sound segment (.matrix) and Sound segment metadata (.txt)) due to the combined data from all platforms.

## 4 Experimental Design, Materials and Methods

The comprehensive nature of using both hands and all ten fingers for recording keyboard sounds is highlighted by combining these ideas. While there may not be a significant difference between left and right-hand typing in terms of sound execution, capturing data from both hands ensures a more thorough dataset, potentially capturing subtle variations in typing behaviour. Employing this method aligns with touch typing techniques commonly utilized by proficient typists for efficient and accurate typing. In touch typing, specific keys are assigned to each finger, following a standardized layout such as the QWERTY layout [7].

Usability, which is a fundamental aspect of data collection, played a critical role in this research. Similar to how user experience is prioritized in the design of different keyboard layouts, like the curved QWERTY layout for smartphones [8], Prioritizing participant comfort throughout data collection was of extreme significance. Providing usability allowed researchers to guarantee the efficacy of their research while also improving participant experience [9]. However, researchers often encounter significant usability challenges when accessing the data [10]. Therefore, researchers investigate various usability techniques during data collection, with the goal of evaluating and achieving optimal data accessibility from the users' perspective. The emphasis on user comfort, even during the collection of keyboard sound data, is consistent with the significance of usability in capturing authentic typing patterns and acquiring high-quality acoustic data.

**Fig. 2:**
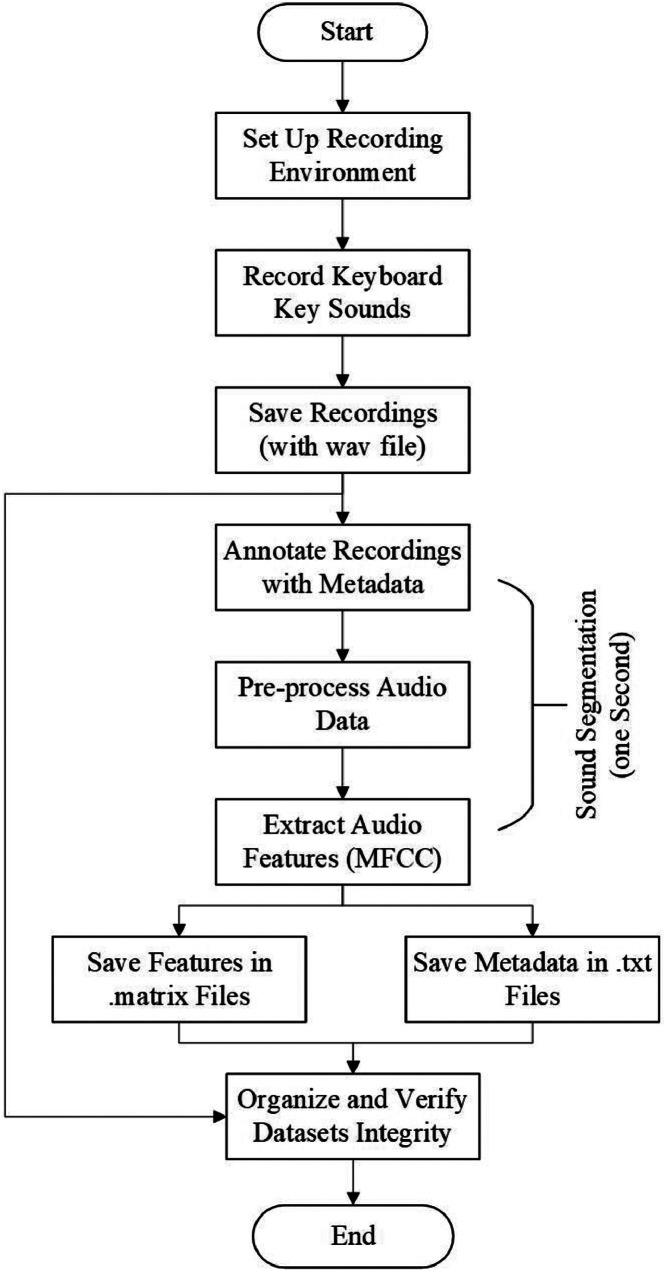
Workflow for building proposed MKA Datasets.

Fig. 2 represents a comprehensive work flow for building a keyboard sound dataset that is crucial for machine learning applications. Rectangular boxes with rounded corners represent each step, connected by arrows to illustrate the flow from “Start” to “End.” Each box clearly describes the stage, like “Record Keyboard Key Sounds” or “Extract Audio Features.” This diagram facilitates understanding the process of building a robust dataset for machine learning.

Overall, this approach demonstrates thoroughness and attention to detail, contributing to the integrity and usefulness of the Multi-Keyboard Acoustic (MKA) Datasets. It captures a comprehensive representation of keyboard sounds, considering variations in typing style and hand positioning among individuals.

### 4.1 Data collection

Recent technological advancements have completely transformed the technique of identifying the sounds produced by keyboard keys. Advanced algorithms and technologies powered by machine learning, deep learning, and artificial intelligence can now be used by researchers to analyse and identify individual keystrokes. These advancements enhance our comprehension of keyboard acoustics, better the precision of sound identification algorithms, and assist in the creation of more reliable and user-friendly human-computer interaction systems.

The data collection was carried out by a group of authors in the Computer Science Department at the University of Halabja. In addition to the main members of this research, three other users from different locations participated in collecting the dataset, particularly for recordings on Zoom and Messenger platforms. These platforms are widely used online communication tools. The participants, aged 19 to 37 years old, included an equal gender balance of four males and four females. They typed in various locations, including a university lab, office, home, and free-space areas. All participants maintained a proper typing posture, sitting close to the keyboard with minimal distance for natural typing conditions.

A multi-datasets of audio recordings of keyboard key sounds were created, collecting high-quality sound from commonly used keyboards. Data was obtained utilizing advanced recording technologies and major platforms such as HP, Lenovo, MSI, Mac, Messenger, and Zoom. The data was acquired in controlled situations using high-quality microphones and sound processing tools to assure consistency and accuracy. Every individual key press was thoroughly documented, and the unprocessed audio data was standardized and saved as wave files for additional examination. This dataset is crucial for doing research on acoustic side-channel attacks and keyboard input recognition systems, emphasizing the significance of meticulous data collecting and inventive recording methods.

Below are the specifications of the four computers and their keyboards:

1. HP ProBook 6570b:
  • CPU:Intel Core i5–3320 m, 2.6GHz
  • RAM: 8192MB
  • Operating System:Windows 10 Pro 64-bit
  • Keyboard: Standard laptop keyboard with membrane switches, providing a soft tactile feedback typical of non-mechanical keyboards.
2. Lenovo Ideapad 110:
  • CPU: Intel Core i7–6498DU, 2.6GHz
  • RAM: 8192MB
  • Operating System: Windows 10 - 64bit
  • Keyboard: Full-sized, chiclet-style keyboard with a numeric keypad. It lacks the tactile feedback of mechanical keyboards but offers a comfortable typing experience.
3. MacBook Pro 2012:
  • CPU: Intel Core i5, 2.5 GHz dual-core (Turbo Boost up to 3.1 GHz) with 3 MB L3 cache
  • RAM: 8GB of 1600 MHz DDR3 memory
  • Operating System: macOS
  • Keyboard: Full-size backlit keyboard with 78 (U.S.) or 79 (ISO) keys, including 12 function keys and 4 arrow keys (inverted “T” arrangement) with an ambient light sensor. Provides a comfortable typing experience with moderate tactile feedback.
4. MSI Pulse GL66 11UEK-034:
  • CPU:Intel Core i7–11800H, 2.30GHz
  • GPU:NVIDIA GeForce RTX 3060 Laptop GPU 6 GB GDDR6
  • RAM: 16 GB
  • Storage: 1 TB HDD + 512 GB NVMe SSD
  • Operating System: Windows 11
  • Keyboard: Standard laptop keyboard, suitable for gaming, with moderate tactile feedback and backlighting.

The following provides a more comprehensive illustration of the recording tools, which are as follows:

1. Microphones Used:
  • Shure MV7: This microphone is chosen for its high sensitivity and ability to capture clear audio, making it suitable for capturing subtle keyboard sounds. It is a dynamic microphone with USB and XLR outputs, designed for speech and vocal recording.
  • Rode NT1: This condenser microphone is known for its low noise and high fidelity, ensuring accurate sound recording. It is ideal for studio-quality audio captures.
  • iPhone 15: We also used the iPhone 15 for recording, leveraging its advanced built-in microphones to capture high-quality audio.
2. Recording Setup:
  • The microphones were placed as close as possible to the device being recorded, within a distance of approximately 10 to 20 cm, to ensure maximum clarity and minimize ambient noise interference.
  • The sampling rate used for our recordings is 44,100 Hz.

Initially, the Shure MV7 and Rode NT1 microphones were used to record audio for letter and number keys, resulting in 36 initial classes. As the project progressed, the dataset was expanded to include >70 classes by incorporating additional keys, such as symbols, beyond just letters and numbers. During this expansion, the iPhone 15′s microphone was also used, particularly in the later stages, adding further diversity to the dataset. This comprehensive approach, as detailed in Table 1, reflects the range of audio conditions and classes captured in our study.

Regarding the hardware used for Zoom and Messenger, we utilized the following keyboards:

1. Dell Inspiron 15 3000 Series:
  • CPU: Intel Core i5–1135G7, 2.4GHz
  • RAM: 8GB DDR4
  • Operating System: Windows 10 Home 64-bit
  • Keyboard: Full-sized chiclet-style keyboard with a numeric keypad. Offers comfortable typing with quiet keys, suitable for everyday use.
2. ASUS VivoBook 15:
  • CPU: AMD Ryzen 5 3500 U, 2.1GHz
  • RAM: 8GB DDR4
  • Operating System: Windows 10 Home 64-bit
  • Keyboard: Full-sized, ergonomic backlit keyboard with chiclet keys. The keyboard is designed for comfortable typing with 1.4 mm key travel, providing a balance of soft tactile feedback and responsive keystrokes.

In order to keep the data quality high, certain rules were put in place during the data collection phase. An exhaustive review was carried out to identify and resolve any discrepancies or inconsistencies in the recorded acoustic components, and exact recording techniques were employed. Trained researchers rigorously evaluated some of the recorded keyboard sounds to increase the dataset's validity. After identifying samples that showed abnormalities or had low quality, they were either re-evaluated or removed from the dataset.

### 4.2 Sound segmentation and pre-processing

The collected data is processed and divided into segments using the Praat program, a highly adaptable tool for acoustic analysis [11]. This system provides a wide range of methods, including spectrographic evaluations and neural networks. By partitioning the original signal spectrum into fragments and selecting one second from each segment displaying a prominent intensity signal, the datasets are optimized for subsequent analysis. Recording 30 clicks in approximately 15 s for each key press allows for a robust representation of the sound produced by each key. Segmenting the recorded WAV files into six separate one-second files demonstrates meticulous attention to detail and ensures the datasets are appropriately structured for analysis. Pre-processing the data with Praat to select the best signal further enhances the quality and reliability of the datasets.

The selected audio is subsequently saved to a file with a .wav extension. To get started with the Praat software, the following step entails accessing the wave file and transforming it into an MFCC file through spectrum analysis. As the final step in making one second of sound, the MFCC file is converted into matrix format and meta-data with (.txt) format.

This study utilized a unique methodology for the annotation and pre-processing of data, with a particular focus on analysing keyboard sounds. Instead of employing Python code for the tasks of labelling and pre-processing, it was decided to apply manual annotation using Praat, a software tool specifically designed for acoustic analysis. This decision enabled the authors to acquire a more thorough and accurate comprehension of the keyboard sound data. By employing this method, they achieved the ability to categorize and enhance annotations with enhanced precision, effectively capturing the subtleties linked to keyboard sound characteristics.

### 4.3 Labelling

Dataset labelling is an essential step in creating new datasets, as it includes providing meaningful and precise labels to the data encompassed within. This method involves the systematic arrangement or grouping of data based on particular criteria or attributes. A primary directory named “MKA Datasets” structures the MKA Datasets, containing seven subdirectories: six for each platform separately (HP, Lenovo, MSI, Mac, Messenger, and Zoom), and one for aggregated data from all platforms. Each platform's directory has four subsidiary folders: Raw Data, Sound Segment (wav), Sound Segment (.matrix), and Sound Segment metadata (.txt). Having both (.txt) and (.matrix) files for each segment of wave sound in the collection of keyboard sound datasets is pivotal. The (.txt) files provide metadata like recording environment and keystroke intensity, guiding pre-processing. Meanwhile, the (.matrix) files hold numerical representations crucial for machine learning model training, capturing features like frequency components and spectrogram data. During training, (.txt) files inform pre-processing, while (.matrix) files supply numerical features for model input, enabling the construction of accurate models for tasks such as keyboard sound classification or activity recognition.

The Sound folder includes audio recordings of keyboard key presses, meticulously arranged into separate folders for each key classification. The Raw Data folder produces six WAV files from the corresponding raw files in each class folder. The files are methodically labelled using the format “class_name+1” to “class_name+6.” The Matrix folder contains files that have been converted from segmented sound files into matrix format. Each file is titled using the same labelling scheme, but with a “matrix” and “txt” extension. This methodical labelling approach ensures a particular arrangement of the dataset, facilitating streamlined data retrieval and analysis.

## Limitations

In the process of compiling the Multi-Keyboard Acoustic (MKA) Datasets, we confronted several methodological limitations. Primarily, challenges emerged during data collection, stemming from variations inherent in recording environments and equipment setups, potentially leading to inconsistencies within the dataset. Furthermore, due to practical constraints, the dataset's scale may not fully encompass the complexity of real-world scenarios, thereby imposing constraints on the generalization potential of machine learning models trained on this dataset. Additionally, a notable consideration involves potential biases inherent in the dataset composition, which may skew towards particular keyboard models or platforms, consequently influencing model performance across diverse devices. Despite efforts to mitigate these issues through rigorous noise reduction and pre-processing techniques, residual noise from recording environments and disparities in microphone characteristics persist as inherent limitations. While our dataset primarily captures clean keyboard sounds, it does not comprehensively account for more complex scenarios such as typing with background noise, including conversations or environmental sounds. Although we provided raw data that may contain such instances, the segmentation process was designed to prioritize signal clarity. Future iterations of the MKA Dataset could benefit from incorporating more varied and noisy environments to enhance the generalizability of machine learning models in real-world conditions. Notwithstanding these constraints, the MKA Datasets stands as a significant scholarly resource, offering critical insights into the realm of keyboard sound recognition across varied platforms.

## Ethics Statement

The authors confirm that they have read and adhere to the ethical requirements for publication in Data in Brief. The present work, which involves the creation of the Multi-Keyboard Acoustic (MKA) Datasets, does not involve human subjects, animal experiments, or data collected from social media platforms. All data were collected and processed in a manner that ensures compliance with established ethical standards. The communication data recorded via Zoom and Messenger platforms were created solely between the authors of this paper, ensuring no involvement of outside individuals. This ensures the integrity and ethical responsibility of the research conducted.

## CRediT Author Statement

**Karwan M. Hama Rawf:** Conceptualization, Data curation, Original draft preparation, Software. **Ayub O. Abdulrahman:** Data curation, Writing- Reviewing. **Hana O. Kamel:** Visualization, Data curation, Investigation Writing- Reviewing and Editing. **Ahmad O. Ali:** Data curation, Methodology. **Lawen M. Hassan:** Validation, Software.

## Data Availability

Multi-Keyboard Acoustic (MKA) Datasets (Original data) (Mendeley Data)
